# Mental health in pandemic times - a review

**DOI:** 10.1192/j.eurpsy.2021.832

**Published:** 2021-08-13

**Authors:** D. Jeremias, A. Moura, D. Rodrigues, C. Laginhas, J. Isaac, R. Albuquerque

**Affiliations:** 1 Psychiatry Department, Ocidental Lisbon Hospital Center, Lisboa, Portugal; 2 Psychiatry, ULSBA - Hospital José Joaquim Fernandes, Beja, Portugal

**Keywords:** COVID-19, psychiatry, self-isolation, telepsychiatry

## Abstract

**Introduction:**

Any outbreak of pandemic dimension will most likely produce a serious amount of distress and prejudice to anyone, in particular when it comes to mental health. The pandemic impact in primary care and in the psychiatric emergency department are some of the topics discussed in this review.

**Objectives:**

It aims to review, evaluate and reflect over the impact of a deadly coronavirus pandemic on mental health, as well as presenting possible long-term challenges and potential ways to approach it.

**Methods:**

A non-systematic literary review was performed on the Pubmed, PsycInfo and Cochrane databases using the key words “covid-19”, “psychiatry”, “self-isolation” and “telepsychiatry”.

**Results:**

Globally and, as expected, there has been a general increase in need for psychiatric assessment and treatment due to the COVID-19 pandemic.

**Conclusions:**

The role of psychiatry has faced quite some challenges in such a short period of time: the rise of telepsychiatry; the management of patients with both a psychiatric disorder and an infection with the new coronavirus and the need to provide an adequate psychiatric assistance in the emergency room has become the new normal.

